# Early supplement of probiotics reduces the risk of obesity among preschool children: a real-world observational study

**DOI:** 10.3389/fnut.2025.1597894

**Published:** 2025-05-15

**Authors:** Maolin Zhang, Liwen Ding, Esben Strodl, Xiaona Yin, Guomin Wen, Dengli Sun, Danxia Xian, Yafen Zhao, Yuxing Zheng, Feitong Liu, Ruibiao Hu, Lingling Zhao, Weikang Yang, Weiqing Chen

**Affiliations:** ^1^Department of Epidemiology and Health Statistics, School of Public Health, Sun Yat-sen University, Guangzhou, China; ^2^School of Psychology and Counselling, Queensland University of Technology, Brisbane, QLD, Australia; ^3^Women’s and Children’s Hospital of Longhua District of Shenzhen, Shenzhen, China; ^4^Biostime (Guangzhou) Health Products Ltd., Guangzhou, China

**Keywords:** probiotics, overweight, obesity, preschooler, nutritional supplement

## Abstract

**Introduction:**

Recent studies have mainly focused on the relationship between probiotic supplementation and childhood obesity in infancy and school-age periods, with a lack of research on preschool stage (3–7 years). This study aimed to explore whether early childhood supplementation with probiotics (0–3 years) could reduce the risk of overweight and obesity among preschoolers.

**Methods:**

A cross-sectional survey was conducted in 2022 among preschoolers from Longhua District, Shenzhen, China. Their mothers were asked to complete a structured questionnaire regarding socio-demographic details and probiotic supplementation of children during the first 0–3 years. Trained professionals measured the children’s current weight and height. Childhood obesity was defined as the body mass index (BMI) being at or above the cut-offs for age and sex according to the BMI growth curves for Chinese children. Multinomial logistic regression analysis was conducted to explore the relationship between probiotic supplementation in children aged 0–3 years and preschool overweight and obesity with controlling for potential confounders. Sex differences, gestational age differences, and birth weight differences were analyzed.

**Results:**

Among the 31,190 children included, 1,389 were classified as obese and 4,337 as overweight. After controlling for potential confounding factors, multinomial logistic regression analysis suggested that probiotic supplementation during the period of age 0–3 years was associated with a lower likelihood of being overweight (AOR = 0.88, 95% CI = 0.82 ~ 0.95) or obesity (AOR = 0.82, 95% CI = 0.72 ~ 0.93). Children who consumed a probiotic product containing *Bifidobacterium longum* subsp*. infantis* R0033, *Bifidobacterium bifidum* R0071, and *Lactobacillus helveticus* R0052 had a lower risk of being overweight (AOR = 0.88, 95% CI = 0.80 ~ 0.96) or obese (AOR = 0.85, 95% CI = 0.73 ~ 0.98). Further stratified analyses showed a significant association with a lower likelihood of obesity only in girls (AOR = 0.70, 95% CI = 0.56 ~ 0.88), but no significant association was observed in boys (AOR = 0.88, 95% CI = 0.75 ~ 1.02).

**Discussion:**

Probiotic supplementation in children aged 0–3 years was associated with a lower risk of overweight and obesity in preschool children, with a potential gender difference. These findings highlight the potential role of early probiotic supplementation in children for preventing overweight and obesity.

## 1 Introduction

Childhood obesity is a serious public health problem worldwide, and its prevalence is increasing at an alarming rate. A recent report indicated that the total number of overweight and obese children below 5 years surpassed 39 million worldwide by 2020 (1). The prevalence of obesity varied widely across countries and regions, and the prevalence of obesity in Chinese children was estimated at 7.77% (95% CI = 7.11 ~ 8.45) (2). Childhood obesity is recognized as a multifactorial metabolic disease (3). The complex interactions between genetic, hormonal, and environmental processes are associated with weight gain (4).

With the global prevalence of childhood obesity, new treatment strategies are being developed. In addition to traditional treatment options, such as dietary control and physical exercise (5), researchers are also exploring the microbiome as a potential approach to treat obesity (6). Among them, prebiotics and probiotics are promising microbiome-based strategies for the prevention and management of obesity in early childhood and the preschool years.

Prebiotics are substances that cannot be digested or absorbed by the host but can promote the growth or activity of beneficial gut microbiota (7). By selectively stimulating the growth of beneficial bacteria such as *Bifidobacterium* and *Lactobacillus*, prebiotics help improve the balance of the gut microbiota, thereby influencing its composition and activity (8). They contribute to increasing the abundance of beneficial microbes while inhibiting the growth of harmful ones. Additionally, prebiotics can indirectly improve host metabolic health by enhancing gut barrier function, strengthening immune responses, and regulating intestinal pH (9, 10).

Probiotics are live microorganisms which when consumed are capable of promoting the health of the host (11). Consumption of over-the-counter probiotics has increased worldwide in recent years (12), and the use of probiotics has become widespread among the general public (13). In the commercial market, probiotic preparations are available in various forms, such as capsules, suspensions, and powders (14). These different formulations vary in terms of stability, palatability, and their impact on bioavailability and compliance (15–17). However, in population-level studies, detailed information on the specific formulation types is often lacking, which limits the assessment of dose–response relationships or formulation-specific effects.

Many studies have demonstrated the positive effects of some probiotics on the health of infants and young children. For example, randomized controlled trials showed that a probiotic supplement (*Bifidobacterium infantis* R0033, *Bifidobacterium bifidum* R0071, and *Lactobacillus helveticus* R0052) contributed to improving mucosal immunity, digestive (18) and exhibit anti-inflammatory effects function in infants (19). The effect of this probiotic combination on preventing overweight and obesity in children is still unknown, although many clinical trials in humans have provided evidence for the use of other probiotics in the treatment of childhood obesity (20, 21).

Most studies support the use of probiotics in the first year of early childhood to intervene in the obesity process (22–24), but there is less research supporting it in the following 2 years. The Developmental Origins of Health and Disease (DOHaD) hypothesis (25) emphasizes the existence of a specific sensitive period of intrauterine or early childhood development, and from the perspective of this hypothesis, the first 1,000 days of life (from conception to 2 years of age after birth) are considered to be the critical period. The first 1,000 days of life is also a crucial period for the development of the gut microbiota, and it is a window of opportunity for shaping a healthy gut microbiota. Certain gut microorganisms, such as *Bifidobacterium*, *Lactobacillus*, and *Bacteroides*, are widely recognized for their beneficial roles in modulating host metabolism, immunity, and neurodevelopment (26–29). In fecal samples from healthy individuals, the typical concentration of these species ranges from 10^7^ to 10^11^ CFU/g, and maintaining an appropriate abundance within this range is considered critical for their functional efficacy (30–32). Disruption of this balance during early life may increase the risk of metabolic disorders, including obesity.

Before the introduction of complementary foods (0–6 months of age), due to relatively monotonous diet (mainly breast milk or formula milk), the types and quantities of gut microbiota in infants are relatively limited, with low diversity, and *Bifidobacterium* is the predominant species. With the introduction of complementary foods (after 6 months of age), the diversity of gut microbiota increases significantly, and the microbiota structure becomes more complex and gradually approaches that of adults. For example, studies have found that after the introduction of complementary foods, more diverse species of the genera *Lactobacillus* and *Bacteroides* appear in the intestine (33). During this developmental stage, early alterations in the gut microbiota may not only influence neurophysiological processes through the bidirectional communication of the gut-brain axis, potentially regulating the production and modulation of neurotransmitters within the enteric nervous system and affecting children’s neurodevelopment (34, 35), but also impact the development of childhood obesity by modulating nutrient absorption and metabolism (36). As such, there is a need to explore whether the period from 0 to 6 months is a sensitive window for probiotic intervention, whether the period after 6 months is more critical, or whether the timing of probiotic supplementation within the first 3 years of life does not make a significant difference. Moreover, while some studies have examined the differential effects of probiotics on obesity prevention and treatment in adults of different genders (37), there is a lack of research on the differences in children of varying sex, gestational ages and birth weights.

The aim of this study was to investigate the relationship between probiotic supplementation during the age of 0–3 years and preschool overweight and obesity. We hypothesize that: (1) probiotic supplementation during the age of 0–3 years is a protective factor against preschool overweight and obesity; and (2) there were sex, gestational age and birth weight differences in the protective effect of probiotic supplementation against childhood overweight and obesity.

## 2 Methods

### 2.1 Study design and participants

**Figure 1:**
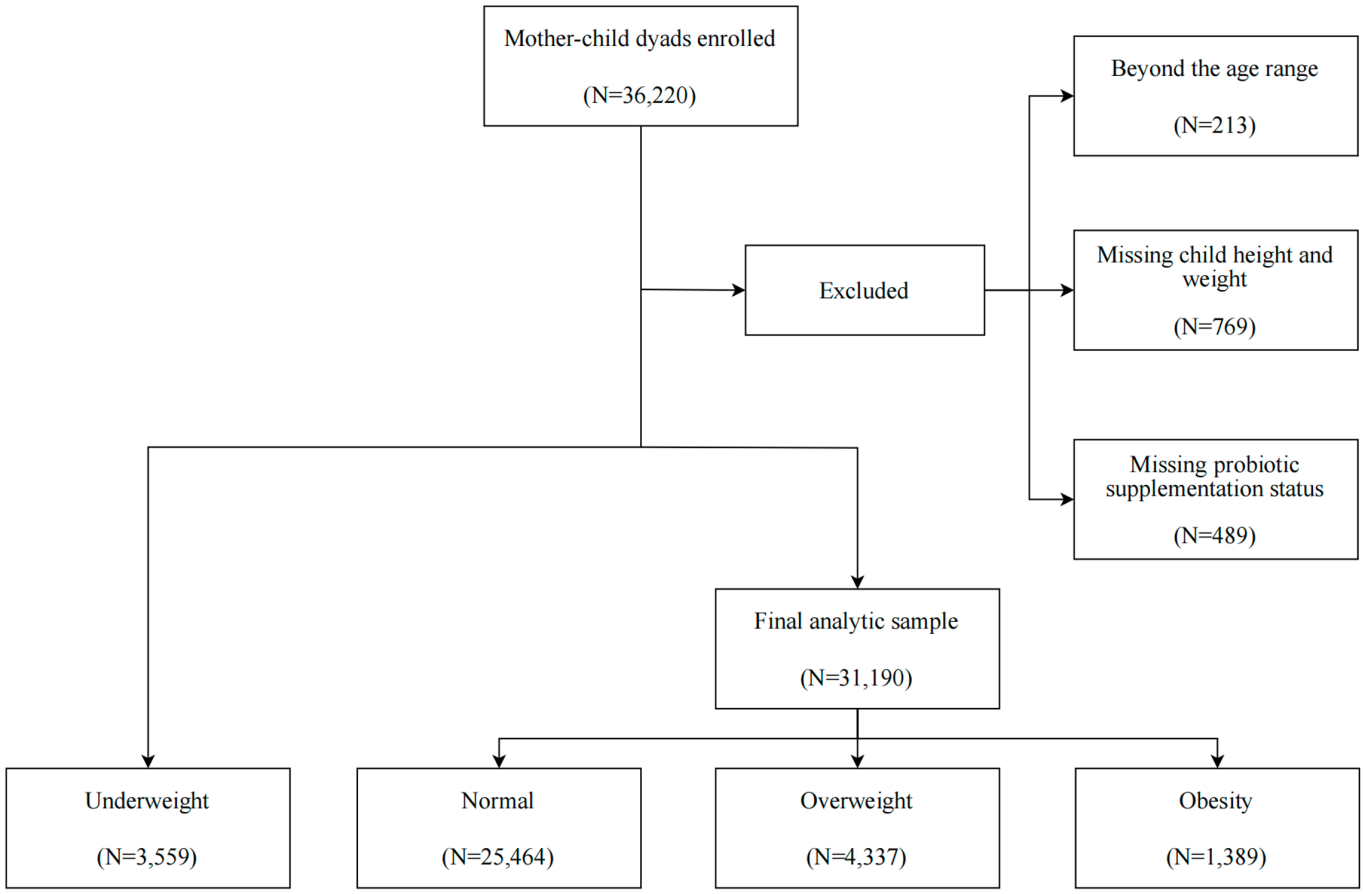
Flow chart of the analytic sample selection process.

A population-based survey was conducted in a total of 36,220 preschool children (3–7 years) from November until December 2022 in 240 registered kindergartens in the Longhua District of Shenzhen, China. The data used in this study are considered real-world data (RWD), as they were obtained from a community-based survey conducted in routine kindergarten settings. We excluded children based on the following criteria: (1) a lack of information on probiotic supplementation for ages 0–3; (2) missing or incorrect data on height and weight; (3) children with severe physical illnesses and mental disorders (Figure 1). After these exclusions, we ultimately included 31,190 children in the final data analysis. Within this sample, we utilized multiple imputation (MI) to estimate the missing data for covariates among questionnaires that were missing information on at least one selected covariate. The study was approved by the Ethic Committee of the Maternal & Child Healthcare Hospital in Longhua District, and the written informed consent was obtained from the mothers of all the children involved in the study, in accordance with the Declaration of Helsinki.

### 2.2 Data collection

The well-trained doctors in the kindergartens directed the mothers to complete a self-administered structured questionnaire for collecting the following information: (1) the socio-demographic characteristics of the parents, including age at conception, education level, household income and marital status; (2) parental-related information, including smoking habits, alcohol consumption, and psychological state; (3) pregnancy-related information, including the method of conception (natural or assisted reproduction) and complications during the pregnancy; (4) birth-related variables, including gestational age and birth weight; (5) the child’s birth date and gender; (6) maternal recall of feeding pattern (breastfeeding, bottle feeding and mixed feeding), child nutritional status at 1–3 years old (general, poor-nourished or well-nourished), child physical activity frequency at 1–3 years old (0, 1–3, 4–6, 7 days/week) and child sleep duration at 1–3 years old.

### 2.3 Probiotic supplementation measurement

The mothers of children were asked the following questions regarding the probiotic supplementation of children during ages 0–3 years: (1) “Was the child supplemented with probiotics during the age of 0–3 years?” (Two response options: “No” or “Yes”); (2) “What was the commencement time of probiotic intake for the child during the ages of 0–3 years” (Five response options: “Not taken,” “0–6 months,” “6–12 months,” “12–36 months,” “Not clear”); (3) “What was the cumulative duration of probiotic intake for the child during the age of 0–3 years”: (Six response options: “Not taken,” “Less than 1 month,” “1–3 months,” “3–6 months,” “More than 6 months,” “Not clear”); (4) “What was the main product of probiotics the child primarily took during the ages of 0–3 years”: (Five response options: “Not taken,” “Product A,” “Product B,” “Other products,” “Not clear”). The probiotic strains contained in each product are presented in Supplementary Table S1.

### 2.4 Measurement and definition of obesity

At Longhua Maternal and Child Health Hospital, trained nurses conducted standardized measurements of each child’s height and weight. The weight was measured using a portable electronic scale with a precision of 0.01 kg, placed on a flat surface. Each preschooler was required to stand in the center of the scale, bareheaded, barefoot, and dressed in light, form-fitting clothing. Measurements were recorded once the scale readings stabilized, with values rounded to the nearest 0.1 kg. Height was measured using a human column altimeter, with a fractional precision of 0.1 cm. The altimeter was positioned vertically against a wall on a horizontal floor. Each child was instructed to stand barefoot and bareheaded on the base, with heels together, feet at a 60-degree angle, chest straight, stomach tucked, and eyes directed forward. Nurses then recorded the height by adjusting the slider to the apex of the child’s head, ensuring the measurement was taken at the highest point of the skull.

Body mass index (BMI) was calculated by dividing weight in kilograms by the square of height in meters (kg/m^2^). Childhood overweight and obesity were defined as a BMI ≥ the relevant cut-offs for age and sex, and childhood underweight was defined as a BMI < the cut-offs for age and sex according to the BMI growth curves for Chinese children (Table 1). These curves are the standardized growth curve for BMI in children under 7 years of age in China, developed using the LMS method by the Capital Institute of Pediatrics (38). The fundamental assumption of the LMS method is that, after a Box-Cox power transformation, the data at each age follow a normal distribution (39). The method summarizes the data using three smooth age-specific curves: L (lambda), M (mu), and S (sigma). The M and S curves represent the median and the coefficient of variation of BMI at each age, while the L curve accounts for the significant age-dependent skewness in the distribution of BMI.

**Table 1:** BMI cut-off points for underweight, overweight and obesity in children aged 3–7 years (kg/m^2^).

Age (years)	Boys			Girls		
	Underweight	Overweight	Obesity	Underweight	Overweight	Obesity
3.0	14.0	16.8	18.1	13.7	16.9	18.3
3.5	13.8	16.6	17.9	13.5	16.8	18.2
4.0	13.6	16.5	17.8	13.4	16.7	18.1
4.5	13.5	16.4	17.8	13.3	16.6	18.1
5.0	13.4	16.5	17.9	13.2	16.6	18.2
5.5	13.4	16.6	18.1	13.1	16.7	18.3
6.0	13.4	16.8	18.4	13.0	16.7	18.4
6.5	13.3	17.0	18.8	13.0	16.8	18.6
7.0	13.4	17.2	19.2	12.9	16.9	18.8

### 2.5 Potential confounding variables

According to previous literature (40–48), and based upon the feasibility of data collection, a range of potential confounders that could impact a child’s BMI were controlled for. These included the child’s gender and age, gestational age, birth weight, maternal age at conception and pre-pregnancy BMI, parental education level and marital status, household income, the child’s feeding pattern, physical activity frequency and electronic screen usage.

### 2.6 Statistical analysis

Descriptive analyses were stratified by child weight status. Categorical variables were presented as frequencies and percentages. Differences within groups were tested using the Chi-squared test for categorical variables. The detailed Chi-squared test results are provided in Supplementary Table S2.

Multinomial logistic regression models were used to evaluate the association of probiotic supplementation status with childhood overweight and obesity statuses after adjusting for the potential confounding variables. The dependent variable was a categorical variable of body weight: normal weight (reference = 0), overweight (case = 1) and obese (case = 2). Probiotic supplementation status includes: (1) whether or not the probiotic was supplemented; (2) when supplementation was initiated; (3) the cumulative duration of supplementation; (4) the primary strains. All regression models were adjusted for the potential confounding variables with adjusted OR (AOR) and 95% CI calculated. Simplified binary logistic regression model is presented in Supplementary Tables S2, S3. To assess the impact of geographic factors on the study results, we included kindergarten as a random effect and performed Bayesian multinomial logistic regression, as presented in Supplementary Table S4. Additionally, we conducted stratified analyses by sex, gestational age and birth weight to evaluate potential difference in the effect of whether probiotics have been consumed on childhood obesity. For each stratification variable (sex, gestational age, and birth weight), we conducted multiple comparisons and applied the Bonferroni correction to adjust the significance level.

All statistical analyses were performed using R version 4.2.3 (R Foundation for Statistical Computing, Vienna, Austria). All two-sided *p*-values < 0.05 were considered statistically significant.

## 3 Results

### 3.1 Descriptive statistics stratified according to child weight status

Table 2 presents the characteristics of the study participants stratified by different child weight status. Compared to normal weight children, obese children are more likely to be boys (70.6% vs. 50.5%), to be born prematurely (7.6% vs. 7.1%), to be macrosomia (4.9% vs. 2.2%), to have mothers with older pregnancies (11.9% vs. 9.9%), and to have mothers with larger pre-pregnancy BMI (15.7% vs. 9.0%).

**Table 2:** Descriptive statistics stratified according to child weight status.

Characteristics	Total number	Normal weight (*n*, %)	Overweight (*n*, %)	Obesity (*n*, %)	*p*-value^a^
Gender					<0.001
Male	16,504	12,864 (50.5)	2,660 (61.3)	980 (70.6)	
Female	14,686	12,600 (49.5)	1,677 (38.7)	409 (29.4)	
Age (years)					0.006
3 ~ 4	7,540	6,238 (24.5)	988 (22.8)	314 (22.6)	
4 ~ 5	10,096	8,145 (32.0)	1,504 (34.7)	447 (32.2)	
5 ~ 6	9,944	8,107 (31.8)	1,379 (31.8)	458 (33.0)	
6 ~ 7	3,610	2,974 (11.7)	466 (10.7)	170 (12.2)	
Gestational age (weeks)					0.261
<37	2,246	1805 (7.1)	336 (7.7)	105 (7.6)	
≥37	28,944	23,659 (92.9)	4,001 (92.3)	1,284 (92.4)	
Child birth weight (g)					<0.001
<2,500	2,129	1781 (7.0)	260 (6.0)	88 (6.3)	
2,500 ~ 4,000	28,254	23,118 (90.8)	3,903 (90.0)	1,233 (88.8)	
>4,000	807	565 (2.2)	174 (4.0)	68 (4.9)	
Maternal age at conception (years)					0.003
<35	28,005	22,934 (90.1)	3,847 (88.7)	1,224 (88.1)	
≥35	3,185	2,530 (9.9)	490 (11.3)	165 (11.9)	
Maternal pre-pregnancy BMI (kg/m^2^)					<0.001
<18.5	6,092	5,247 (20.6)	675 (15.6)	170 (12.2)	
18.5 ~ 23.9	22,008	17,916 (70.4)	3,091 (71.3)	1,001 (72.1)	
≥24	3,090	2,301 (9.0)	571 (13.2)	218 (15.7)	
Maternal education level					0.008
≤Middle school	3,494	2,782 (10.9)	527 (12.2)	185 (13.3)	
High school	5,362	4,356 (17.1)	751 (17.3)	255 (18.4)	
College	21,081	17,314 (68.0)	2,870 (66.2)	897 (64.6)	
≥Postgraduate	1,253	1,012 (4.0)	189 (4.4)	52 (3.7)	
Paternal education level					<0.001
≤Middle school	3,134	2,511 (9.9)	447 (10.3)	176 (12.7)	
High school	5,551	4,424 (17.4)	847 (19.5)	280 (20.2)	
College	20,634	17,018 (66.8)	2,770 (63.9)	846 (60.9)	
≥Postgraduate	1871	1,511 (5.9)	273 (6.3)	87 (6.3)	
Household income (RMB/month)					0.848
≤10,000	4,680	3,804 (14.9)	652 (15.0)	224 (16.1)	
10,001 ~ 20,000	9,705	7,928 (31.1)	1,347 (31.1)	430 (31.0)	
20,001 ~ 30,000	6,615	5,379 (21.1)	939 (21.7)	297 (21.4)	
>30,000	10,190	8,353 (32.8)	1,399 (32.3)	438 (31.5)	
Feeding pattern					0.985
Breastfeeding	17,432	14,243 (55.9)	2,410 (55.6)	779 (56.1)	
Bottle feeding	3,248	2,642 (10.4)	464 (10.7)	142 (10.2)	
Mixed feeding	10,394	8,485 (33.3)	1,445 (33.3)	464 (33.4)	
Not clear	116	94 (0.4)	18 (0.4)	4 (0.3)	
Child physical activity frequency (days/week)					0.902
0	81	64 (0.3)	12 (0.3)	5 (0.4)	
1 ~ 2	6,764	5,542 (21.8)	939 (21.6)	283 (20.4)	
3 ~ 6	10,536	8,591 (33.7)	1,464 (33.8)	481 (34.6)	
7	13,809	11,267 (44.2)	1922 (44.3)	620 (44.6)	
Electronic screen usage (minutes/day)					0.007
0	7,572	6,223 (24.4)	994 (22.9)	355 (25.6)	
1 ~ 30	14,679	12,009 (47.2)	2063 (47.6)	607 (43.7)	
31 ~ 60	6,692	5,452 (21.4)	932 (21.5)	308 (22.2)	
>60	2,247	1780 (7.0)	348 (8.0)	119 (8.6)	

^a^*P*-values were obtained using the Chi-squared test.

### 3.2 Probiotic supplementation of children with different body weight

The probiotic supplementation of children with different body weight is shown in Table 3. The results indicate that for the combine weight statuses 24,435 (78.3%) children consumed probiotics within the first 3 years of their life, while 6,755 (21.7%) children did not. Regarding the initiation time of probiotic supplementation, 7,678 (24.6%) children taken probiotics within the 12–36 months, constituting the highest proportion among all age groups. Concerning the cumulative duration of probiotic consumption, 8,496 (27.2%) children had a cumulative intake duration of less than 1 month, which represented the highest frequency across all duration categories. Obese children were more likely not to take probiotics than normal weight children (25.1% vs. 21.2%).

**Table 3:** Probiotic supplementation stratified by child weight status.

Probiotic supplementation	Total number	Normal weight (*n*, %)	Overweight (*n*, %)	Obesity (*n*, %)	*p*-value^a^
Whether taking					<0.001
No	6,755	5,394 (21.2)	1,013 (23.4)	348 (25.1)	
Yes	24,435	20,070 (78.8)	3,324 (76.6)	1,041 (74.9)	
Initiation time (months)					<0.001
No	6,755	5,394 (21.2)	1,013 (23.4)	348 (25.1)	
0–6	5,037	4,120 (16.2)	722 (16.6)	195 (14.0)	
6–12	7,607	6,239 (24.5)	1,025 (23.6)	343 (24.7)	
12–36	7,678	6,360 (25.0)	991 (22.8)	327 (23.5)	
Not clear	4,113	3,351 (13.2)	586 (13.5)	176 (12.7)	
Cumulative duration (months)					<0.001
No	6,755	5,394 (21.2)	1,013 (23.4)	348 (25.1)	
<1	8,496	7,067 (27.8)	1,079 (24.9)	350 (25.2)	
1–3	4,697	3,843 (15.1)	660 (15.2)	194 (14.0)	
3–6	2,704	2,216 (8.7)	377 (8.7)	111 (8.0)	
>6	4,021	3,277 (12.9)	566 (13.1)	178 (12.8)	
Not clear	4,517	3,667 (14.4)	642 (14.8)	208 (15.0)	
Probiotic product					0.003
No	6,755	5,394 (21.2)	1,013 (23.4)	348 (25.1)	
Product A	8,701	7,127 (28.0)	1,183 (27.3)	391 (28.1)	
Product B	4,695	3,853 (15.1)	644 (14.8)	198 (14.3)	
Other strains	3,535	2,932 (11.5)	465 (10.7)	138 (9.9)	
Not clear	7,504	6,158 (24.2)	1,032 (23.8)	314 (22.6)	

^a^*P*-values were obtained using the Chi-squared test.

### 3.3 Relationship of probiotic supplementation with overweight and obesity among preschoolers

Table 4 shows the results of multinomial logistic regressions of associations between probiotic supplementation status with children overweight and obesity. After adjusting for potential confounders, the multinomial logistic regressions showed that probiotic supplementation during ages 0–3 years was associated with a lower risk of overweight (AOR = 0.88, 95% CI = 0.82 ~ 0.95) and obesity (AOR = 0.82, 95% CI = 0.72 ~ 0.93) compared to children without probiotic supplementation.

**Table 4:** Associations between probiotic supplementation and children overweight and obesity.

Probiotic supplementation	Overweight (vs. Normal weight)		Obesity (vs. Normal weight)	
	AOR (95% CI)^a^	*p*-value	AOR (95% CI)^a^	*p*-value
Whether taking				
No	ref		ref	
Yes	0.88 (0.82, 0.95)	0.002	0.82 (0.72, 0.93)	0.002
Initiation time (months)				
No	ref		ref	
0–6	0.95 (0.85, 1.05)	0.306	0.76 (0.64, 0.92)	0.004
6–12	0.88 (0.80, 0.97)	0.007	0.87 (0.74, 1.02)	0.075
12–36	0.82 (0.75, 0.91)	<0.001	0.80 (0.68, 0.93)	0.005
Not clear	0.93 (0.84, 1.05)	0.234	0.82 (0.68, 0.99)	0.042
Cumulative duration (months)				
No	ref		ref	
<1	0.82 (0.75, 0.90)	<0.001	0.80 (0.68, 0.93)	0.004
1–3	0.91 (0.82, 1.01)	0.079	0.78 (0.65, 0.94)	0.009
3–6	0.91 (0.80, 1.03)	0.134	0.79 (0.63, 0.98)	0.033
>6	0.91 (0.81, 1.02)	0.088	0.84 (0.69, 1.01)	0.063
Not clear	0.94 (0.84, 1.04)	0.227	0.89 (0.74, 1.06)	0.183
Probiotic product				
No	ref		ref	
Product A	0.88 (0.80, 0.96)	0.005	0.85 (0.73, 0.98)	0.028
Product B	0.91 (0.81, 1.01)	0.079	0.84 (0.70, 1.01)	0.057
Other products	0.85 (0.75, 0.96)	0.008	0.75 (0.61, 0.92)	0.006
Not clear	0.89 (0.81, 0.98)	0.018	0.80 (0.69, 0.94)	0.007

^a^Adjusted for children’s gender and age, gestational age birth weight, maternal age at conception and pre-pregnancy BMI, parental education level and marital status, household income, the child’s feeding pattern, physical activity frequency and electronic screen usage in models. Ref, reference.

Compared to children without probiotic supplementation, those taking probiotics when 6–12 months (AOR = 0.88, 95% CI = 0.80 ~ 0.97) or 12–36 months (AOR = 0.82, 95% CI = 0.75 ~ 0.91) had lower odds of being overweight; while children taking probiotics when 0–6 months (AOR = 0.76, 95% CI = 0.64 ~ 0.92) or 12–36 months (AOR = 0.80, 95% CI = 0.68 ~ 0.93) had lower odds of being obesity.

In comparison to children without probiotic supplementation, those who cumulatively took probiotics for less than 1 month (AOR = 0.82, 95% CI = 0.75 ~ 0.90) had a significantly lower odds of being overweight as a preschooler; while children who consumed probiotics for less than 1 month (AOR = 0.80, 95% CI = 0.68 ~ 0.93), 1–3 months (AOR = 0.78, 95% CI = 0.65 ~ 0.94), and 3–6 months (AOR = 0.79, 95% CI = 0.63 ~ 0.98) respectively had lower odds of being obesity.

Compared to the children without probiotic supplementation, children who consumed product A or other probiotic products had lower odds of being overweight or obese, while those who took product B exhibited no significant reduction in the risk of being overweight (AOR = 0.91, 95% CI = 0.81 ~ 1.01) or obese (AOR = 0.84, 95% CI = 0.70 ~ 1.01) as a preschooler.

Table 5 shows the association of probiotic supplementation with childhood obesity stratified by sex, gestational age and birth weight. The results indicated that probiotic supplementation during ages 0–3 years was associated with lower odds of being overweight in boys (AOR = 0.88, 95% CI = 0.80 ~ 0.97, *p* < 0.025) with a non-significant trend in girls (AOR = 0.89, 95% CI = 0.79 ~ 1.00, *p* > 0.025), although the effect sizes were similar for both sexes. In contrast, boys who took probiotic supplements showed a non-significant trend for a reduced risk of obesity (AOR = 0.88, 95% CI = 0.75 ~ 1.02, *p* > 0.025), while girls taking probiotic supplements were significantly less likely to be obese as preschoolers (AOR = 0.70, 95% CI = 0.56 ~ 0.88, *p* < 0.025).

**Table 5:** Associations between probiotic supplementation and children overweight and obesity: stratified by sex, gestational age and birth weight.

Stratification factors	Probiotic supplementation	Overweight (vs. Normal weight)		Obesity (vs. Normal weight)	
		AOR (95% CI)^a^	*p*-value^b^	AOR (95% CI)^a^	*p*-value^b^
Sex status					
Boys					
	No	ref		ref	
	Yes	0.88 (0.80, 0.97)	0.013	0.88 (0.75, 1.02)	0.090
Girls					
	No	ref		ref	
	Yes	0.89 (0.79, 1.00)	0.054	0.70 (0.56, 0.88)	0.002
Gestational age (weeks)					
<37					
	No	ref		ref	
	Yes	0.89 (0.66, 1.19)	0.416	0.75 (0.46, 1.21)	0.242
≥37					
	No	ref		ref	
	Yes	0.88 (0.81, 0.96)	0.002	0.82 (0.72, 0.94)	0.003
Birth weight (g)					
<2,500					
	No	ref		ref	
	Yes	0.66 (0.48, 0.90)	0.010	0.63 (0.38, 1.05)	0.075
2,500–4,000					
	No	ref		ref	
	Yes	0.89 (0.82, 0.96)	0.004	0.82 (0.71, 0.93)	0.003
>4,000					
	No	ref		ref	
	Yes	1.32 (0.87, 1.99)	0.198	1.10 (0.59, 2.05)	0.762

^a^Adjusted for children’s gender (removed when gender stratification) and age, gestational age (removed when gestational age stratification), birth weight (removed when birth weight stratification), maternal age at conception and pre-pregnancy BMI, parental education level and marital status, household income, the child’s feeding pattern, physical activity frequency and electronic screen usage in models. ^b^The *p*-values were adjusted using the Bonferroni correction, with the significance level for the sex and gestational age stratifications set at 0.025 and the significance level for the birth weight stratification set at 0.0083. ref, reference.

Probiotic supplementation was associated with a lower risk of both overweight (AOR = 0.88, 95% CI = 0.81 ~ 0.96, *p* < 0.025) or obesity (AOR = 0.82, 95% CI = 0.72 ~ 0.94, *p* < 0.025) only in full-term children. Similarly, probiotic supplementation was associated with a lower risk of overweight (AOR = 0.89, 95% CI = 0.82 ~ 0.96, *p* < 0.0083) and obesity (AOR = 0.82, 95% CI = 0.71 ~ 0.93, *p* < 0.0083) only in the normal birth weight group. Nonetheless, these findings should be interpreted with caution due to the limited sample size.

## 4 Discussion

To the best of our knowledge, this is the first study examining the effects of probiotic supplementation during the ages of 0–3 years on overweight and obesity in Chinese preschoolers. Our study identified a significant association between probiotic supplementation in children aged 0–3 years and a lower prevalence of both overweight and obesity in preschoolers. Children who consumed a probiotic product containing *B. longum* subsp. *infantis* R0033, *B. bifidum* R0071, and *L. helveticus* R0052 had a lower risk of being overweight or obese. Additionally, we observed a gender difference in the protective effect of probiotic supplementation against childhood obesity, with girls showing the lower risk of obesity compared to boys. Concurrently, exploratory research indicated a potentially stronger association between probiotic supplementation and reduced obesity risk among children born full-term or with normal birth weight, though these preliminary findings warrant further validation in adequately powered studies.

In our study, probiotic supplement between 0 and 3 years of age was associated with a lower risk of overweight and obesity in preschool-aged children. These findings are supported by several experimental studies that have investigated the potential mechanisms underlying the role of probiotics in pediatric weight management. For instance, a double-blind, randomized, placebo-controlled trial conducted in China found that a 12-week supplementation with a multi-strain probiotic formula (*Lactobacillus salivarius* AP-32, *Lactobacillus rhamnosus* bv-77, and *Bifidobacterium animalis* subsp. *lactis* CP-9) improved gut microbiota composition, elevated high-density lipoprotein (HDL) and adiponectin levels, and reduced BMI, total cholesterol, low-density lipoprotein (LDL), leptin, and tumor necrosis factor-alpha (TNF-*α*) levels in obese children, suggesting a beneficial effect on lipid metabolism and inflammation (49). Similarly, a randomized controlled trial from Thailand reported improvements in adiposity indices, inflammation, and gut microbiota diversity among children who received *Lactobacillus paracasei* HII01 and *B. animalis* subsp. *lactis* supplements (50).

Our research found that probiotic supplementation initiated at different ages between 0 and 3 years was associated with a lower prevalence of overweight and obesity in children. However, some results were not statistically significant. For example, probiotic supplementation initiated between 0 and 6 months was not significantly associated with a lower prevalence of overweight, while supplementation between 6 and 12 months was not significantly associated with a lower prevalence of obesity. Nevertheless, their effect sizes were similar to those observed at other initiation ages. Early probiotic intervention helps regulate the gut microbiota and may reduce the occurrence of obesity by influencing metabolism, the immune system, and energy balance (26). Our findings suggested that probiotic supplementation initiated at different ages between 0 and 3 years was associated with a lower prevalence of overweight and obesity in preschool children, with no evident sensitive period.

Additionally, we found that probiotic supplementation, particularly when the cumulative duration was less than 1 month for overweight and within 6 months for obesity, was associated with a lower prevalence of these conditions. From a certain perspective, this may be because the duration required to reduce the risk of overweight is shorter than that required to reduce the risk of obesity. Individuals with overweight exhibit better metabolic adaptability and behavioral plasticity compared to those with obesity, which may allow for a shorter intervention period to achieve beneficial effects (51). However, this also raises an important question worth considering and discussing: why would a shorter duration be more effective than a longer one? One possible explanation is that the sample sizes in the longer-duration groups may be smaller, resulting in insufficient statistical power to detect the effect size and significance. Future studies with larger sample sizes are needed to further investigate this finding.

In this study, the probiotic strains contained in product A and product B (Supplementary Table S1) have been suggested to associate with a reduced risk of obesity and overweight in previous research. For example, the metabolic by-products of *B. bifidum* R0071 are positively correlated with the number of viable bacteria reaching colonic fermentation, which positively influences the development of the infant gut microbiota (52). What’s more, *B. longum* subsp. *infantis* R0033 and *B. bifidum* R0071 can specifically metabolize oligosaccharides with different structures in breast milk (53). Studies have shown that R0071 can hydrolyze human milk oligosaccharides outside the cells with the help of cell wall-anchored secreted glycosidases. This hydrolysis process releases monosaccharides and disaccharides, which are then taken up by the cells for metabolism and growth. In contrast, R0033 mainly metabolizes human milk oligosaccharides (HMOs) intracellularly. In *in vitro* experiments, when R0071 and R0033 are co-cultured, the overall number of cell proliferations increases significantly, and the two form a cross-feeding relationship. In addition, the degradation products of HMOs released by R0071 during the metabolism of HMOs can also provide the nutrients required for the growth of other strains, thus promoting their growth. R0071 can form a mutually beneficial symbiotic relationship with other bacterial communities, through the sharing of metabolites in the microbial community, jointly maintaining the stability and balance of the gut microbial ecosystem. *L. helveticus* R0052 has been shown to improve weight gain and other obesity markers (54). Probiotic supplementation with *L. rhamnosus* HN001 during pregnancy has been found to reduce the likelihood of overweight in the children of overweight or obese women at 24 months of age (21). Another study reported that daily intake of 80 mL of probiotic yogurt containing *B. animalis* subsp*. lactis* HN019 led to a reduction in BMI (55). However, our study found that, compared to children without probiotic supplementation, children who consumed product A or other probiotic products had a lower prevalence of overweight or obesity, while those who took product B showed no significant difference in prevalence. This suggests that different combinations of probiotic strains may be associated with varying outcomes in childhood overweight and obesity. Future clinical trials could compare the associations of different probiotic formulations with these outcomes.

We found that the association between probiotics and obesity was not equally strong between boys and girls. Compared to boys, probiotic supplementation in children aged 0–3 years was more commonly associated with a lower prevalence of obesity in preschool girls. Previous studies have also explored the relationship between probiotics and obesity, revealing gender differences. In a double-blind, placebo-controlled randomized trial, the female participants in the probiotic group experienced a significantly weight loss than those in the placebo group (*p* = 0.02), while the male participants in both groups showed similar results (*p* = 0.53) (37). Further research revealed that treatment with *L. rhamnosus* CGMCC1.3724 combined with a calorie-restricted diet resulted in significantly higher weight loss in obese females compared to obese males (56). Gender differences in various characteristics may be potential factors influencing this relationship. First, a study in rats suggested that genes related to appetite regulation, such as proopiomelanocortin, neuropeptide Y, leptin receptor, and agouti-related protein, were expressed differently between males and females (57). Second, there are sex-based differences in the composition of the gut microbiota between males and females, which are, to some extent, a result of the effects of sex hormones (58). Further research is needed to explain the gender-specific responses to probiotic supplementation in children and the mechanisms behind these gender differences.

Due to the limited sample size of preterm, low birth weight, and high birth weight children, our preliminary exploratory analysis suggested that probiotic supplementation may only be associated with a lower risk of overweight or obesity among children who are full-term or of normal birth weight. Compared to full-term infants, preterm infants may have abnormal microbiota, with delayed development of the gut microbiota, which may affect the effectiveness of probiotics (59, 60). Regarding birth weight, low birth weight children are often at higher metabolic and growth risks, such as catch-up growth (61), and probiotics may reduce the incidence of overweight and obesity by modulating the gut microbiota and promoting healthy growth and development. In high birth weight children, probiotic supplementation failed to significantly reduce the risk of overweight or obesity. This may be related to the metabolic characteristics of high birth weight children themselves and other growth and metabolic problems they may face (3, 62), which suggests that different intervention strategies are needed to prevent obesity in such children. Future studies with larger sample sizes are needed to enable the interpretation of these findings.

Our findings are also consistent with the biomedical mechanisms by which probiotics influence obesity. From the very first moment of life, a newborn’s body begins to interact with a variety of microorganisms. Probiotics are microbial agents with immunomodulatory, anti-inflammatory, nutritional and anti-microbial effects (63), and are thought to improve gut health by promoting the growth of beneficial microorganisms. Probiotics are able to reduce intestinal endotoxin levels by stabilizing the permeability of the intestinal barrier and attenuating low-grade inflammatory states (64). Additionally, probiotics may promote non-obesogenic changes in the intestinal environment by altering the fermentation process of dietary polysaccharides, which in turn affects energy harvesting and fat deposition in the host (65). During critical periods of life, probiotic interventions may protect individuals from premature weight gain through both immunomodulatory pathways and energy harvesting mechanisms (66, 67), and this effect is usually limited to the first few years of life, when the gut microbial composition and immune response are still being formed. More mechanisms of probiotics on obesity remain to be elucidated.

Although our study primarily focused on epidemiological associations, we fully acknowledge that early-life probiotic supplementation may influence the development of overweight and obesity through epigenetic regulatory mechanisms (68). For example, *Bifidobacterium lactis* LMG P-28149 and *L. rhamnosus* LMG S-28148 have been shown to increase the abundance of *Firmicutes* and *Bacteroidetes* in the gut microbiota, upregulate the expression of peroxisome proliferator-activated receptor gamma (PPARγ) and lipoprotein lipase, and ameliorate obesity in murine models (69). In addition, prenatal supplementation with specific probiotics may reduce the risk of childhood obesity and excessive weight gain by decreasing DNA methylation levels of key genes such as fat mass and obesity-associated gene (FTO), melanocortin 4 receptor (MC4R), insulin-like growth factor binding protein 1 (IGFBP1), and methionine sulfoxide reductase A (MSRA) (70). In future research, integrating epigenetic data will be crucial to better elucidate the underlying biological mechanisms of our statistical findings.

The findings of this study should be interpreted in light of the following limitations. First, all participants were recruited from Longhua District of Shenzhen, which may lead to selection bias and limit the generalizability of our results, as the use methods of probiotics and strains may vary across different regions. Second, the data on probiotic supplementation for children aged 0–3 years was subjectively recalled by the mothers, which may result in recall bias and social desirability bias. Third, due to the proprietary nature of probiotic product formulations in the consumer market, we were unable to identify the probiotic strains used in products other than product A and product B. Fourth, unfortunately, due to the lack of standardized dosage measurement guidelines in the survey, we were unable to collect precise information on the dosage of probiotics taken by children aged 0–3 years, which may limit our ability to assess the relationship between probiotic use in early childhood and overweight or obesity in preschool children. Fifth, despite including a range of covariates, there were still unmeasured potential confounding variables, such as paternal obesity, parental and children’s diet, etc., that may influence the findings. Sixth, in this cross-sectional study, we did not collect data on whether children aged 0–3 years were overweight or obese, which might limit any conclusions on the causal relationship between probiotic supplementation in early childhood and overweight/obesity in preschool children. Therefore, prospective birth cohort studies are needed to determine their causal relationship. Seventh, we did not measure biological indicators such as inflammatory markers and obesity-related biomarkers, which could provide mechanistic insight into the association between early-life exposures and childhood overweight or obesity. The absence of such data is primarily due to logistical and financial constraints in this large-scale, community-based survey. Future studies with biomarker data collection are warranted to validate and extend our findings.

## 5 Conclusion

In summary, probiotic supplementation in children aged 0–3 years was associated with a lower risk of overweight and obesity in preschool children, with a potential gender difference. Supplementation at any time during this early life period appeared to be beneficial in reducing the likelihood of developing overweight or obesity. These findings suggest a possible protective role of early probiotic use, underscoring the need for further longitudinal and experimental studies to confirm its preventive potential. Nevertheless, the interpretation of dose-specific effects is limited by the lack of detailed data on individual probiotic components, highlighting the need for more targeted research in this area.

## Data Availability

The raw data supporting the conclusions of this article will be made available by the authors, without undue reservation.

## References

[1] Obesity and overweight. Available online at: https://www.who.int/news-room/fact-sheets/detail/obesity-and-overweight (accessed April 24, 2025)

[2] Zhang X Liu J Ni Y Yi C Fang Y Ning Q. Global prevalence of overweight and obesity in children and adolescents: a systematic review and Meta-analysis. JAMA Pediatr. (2024) 178:800. doi: 10.1001/jamapediatrics.2024.1576, PMID: 38856986 PMC11165417

[3] Hoffman DJ Powell TL Barrett ES Hardy DB. Developmental origins of metabolic diseases. Physiol Rev. (2021) 101:739–95. doi: 10.1152/physrev.00002.2020 33270534 PMC8526339

[4] Thomas-Eapen N. Childhood obesity. Prim Care. (2021) 48:505–15. doi: 10.1016/j.pop.2021.04.002, PMID: 34311854

[5] Calcaterra V Rossi V Mari A Casini F Bergamaschi F Zuccotti GV. Medical treatment of weight loss in children and adolescents with obesity. Pharmacol Res. (2022) 185:106471. doi: 10.1016/j.phrs.2022.106471 36174963

[6] Sheykhsaran E Abbasi A Ebrahimzadeh Leylabadlo H Sadeghi J Mehri S Naeimi Mazraeh F. Gut microbiota and obesity: an overview of microbiota to microbial-based therapies. Postgrad Med J. (2023) 99:384–402. doi: 10.1136/postgradmedj-2021-141311, PMID: 37294712

[7] Gibson GR Scott KP Rastall RA Tuohy KM Hotchkiss A Dubert-Ferrandon A. Dietary prebiotics: current status and new definition. Food Sci Technol Bull Funct Foods. (2010) 7:1–19. doi: 10.1616/1476-2137.15880

[8] You S Ma Y Yan B Pei W Wu Q Ding C. The promotion mechanism of prebiotics for probiotics: a review. Front Nutr. (2022) 9:1000517. doi: 10.3389/fnut.2022.1000517, PMID: 36276830 PMC9581195

[9] Włodarczyk M Śliżewska K. Obesity as the 21st century’s major disease: the role of probiotics and prebiotics in prevention and treatment. Food Biosci. (2021) 42:101115. doi: 10.1016/j.fbio.2021.101115

[10] Yoo S Jung SC Kwak K Kim JS. The role of prebiotics in modulating gut microbiota: implications for human health. Int J Mol Sci. (2024) 25:4834. doi: 10.3390/ijms25094834, PMID: 38732060 PMC11084426

[11] Hill C Guarner F Reid G Gibson GR Merenstein DJ Pot B. Expert consensus document. The international scientific Association for Probiotics and Prebiotics consensus statement on the scope and appropriate use of the term probiotic. Nat Rev Gastroenterol Hepatol. (2014) 11:506–14. doi: 10.1038/nrgastro.2014.66, PMID: 24912386

[12] Zavišić G Popović M Stojkov S Medić D Gusman V Jovanović Lješković N. Antibiotic resistance and probiotics: knowledge gaps, market overview and preliminary screening. Antibiot Basel Switz. (2023) 12:1281. doi: 10.3390/antibiotics12081281, PMID: 37627701 PMC10451169

[13] Tegegne BA Kebede B. Probiotics, their prophylactic and therapeutic applications in human health development: a review of the literature. Heliyon. (2022) 8:e09725. doi: 10.1016/j.heliyon.2022.e09725, PMID: 35785237 PMC9240980

[14] Kiepś J Dembczyński R. Current trends in the production of probiotic formulations. Food Secur. (2022) 11:2330. doi: 10.3390/foods11152330, PMID: 35954096 PMC9368262

[15] Krunić TŽ Rakin MB. Enriching alginate matrix used for probiotic encapsulation with whey protein concentrate or its trypsin-derived hydrolysate: impact on antioxidant capacity and stability of fermented whey-based beverages. Food Chem. (2022) 370:130931. doi: 10.1016/j.foodchem.2021.130931, PMID: 34509939

[16] Alsharafani MAM Abdullah T Jabur ZA Hassan AA Alhendi AS Abdulmawjood A. Assessing synergistic effect of Jerusalem artichoke juice and antioxidant compounds on enhanced viability and persistence of bifidobacterium species, palatability, and shelf life. Food Sci Nutr. (2022) 10:1994–2008. doi: 10.1002/fsn3.2815, PMID: 35702306 PMC9179157

[17] Baral KC Bajracharya R Lee SH Han HK. Advancements in the pharmaceutical applications of probiotics: dosage forms and formulation technology. Int J Nanomedicine. (2021) 16:7535–56. doi: 10.2147/IJN.S337427, PMID: 34795482 PMC8594788

[18] Xiao L Gong C Ding Y Ding G Xu X Deng C. Probiotics maintain intestinal secretory immunoglobulin a levels in healthy formula-fed infants: a randomised, double-blind, placebo-controlled study. Benef Microbes. (2019) 10:729–39. doi: 10.3920/BM2019.0025, PMID: 31965842

[19] De Andrés J Manzano S García C Rodríguez JM Espinosa-Martos I Jiménez E. Modulatory effect of three probiotic strains on infants’ gut microbial composition and immunological parameters on a placebo-controlled, double-blind, randomised study. Benef Microbes. (2018) 9:573–84. doi: 10.3920/BM2017.0132, PMID: 29726280

[20] Lyons-Reid J Derraik JGB Kenealy T Albert BB Ramos Nieves JM Monnard CR. Impact of preconception and antenatal supplementation with myo-inositol, probiotics, and micronutrients on offspring BMI and weight gain over the first 2 years. BMC Med. (2024) 22:39. doi: 10.1186/s12916-024-03246-w, PMID: 38287349 PMC10826220

[21] Saros L Vahlberg T Koivuniemi E Houttu N Niinikoski H Tertti K. Fish oil and/or probiotics intervention in overweight/obese pregnant women and overweight risk in 24-month-old children. J Pediatr Gastroenterol Nutr. (2023) 76:218–26. doi: 10.1097/MPG.0000000000003659, PMID: 36705702 PMC9848211

[22] Karlsson Videhult F Öhlund I Stenlund H Hernell O West CE. Probiotics during weaning: a follow-up study on effects on body composition and metabolic markers at school age. Eur J Nutr. (2015) 54:355–63. doi: 10.1007/s00394-014-0715-y, PMID: 24830782

[23] Jeong S. Factors influencing development of the infant microbiota: from prenatal period to early infancy. Clin Exp Pediatr. (2022) 65:439–47. doi: 10.3345/cep.2021.00955, PMID: 34942687 PMC9441613

[24] Eor JY Lee CS Moon SH Cheon JY Pathiraja D Park B. Effect of probiotic-fortified infant formula on infant gut health and microbiota modulation. Food Sci Anim Resour. (2023) 43:659–73. doi: 10.5851/kosfa.2023.e26, PMID: 37484007 PMC10359846

[25] Aris IM Fleisch AF Oken E. Developmental origins of disease: emerging prenatal risk factors and future disease risk. Curr Epidemiol Rep. (2018) 5:293–302. doi: 10.1007/s40471-018-0161-0, PMID: 30687591 PMC6345523

[26] Chandrasekaran P Weiskirchen S Weiskirchen R. Effects of probiotics on gut microbiota: an overview. Int J Mol Sci. (2024) 25:6022. doi: 10.3390/ijms25116022, PMID: 38892208 PMC11172883

[27] Saturio S Nogacka AM Alvarado-Jasso GM Salazar N De Los Reyes-Gavilán CG Gueimonde M. Role of bifidobacteria on infant health. Microorganisms. (2021) 9:2415. doi: 10.3390/microorganisms9122415, PMID: 34946017 PMC8708449

[28] Un-Nisa A Khan A Zakria M Siraj S Ullah S Tipu MK. Updates on the role of probiotics against different health issues: focus on lactobacillus. Int J Mol Sci. (2022) 24:142. doi: 10.3390/ijms24010142, PMID: 36613586 PMC9820606

[29] Zafar H Saier MH. Gut bacteroides species in health and disease. Gut Microbes. (2021) 13:1–20. doi: 10.1080/19490976.2020.1848158 PMC7872030 33535896

[30] Tufail MA Schmitz RA. Exploring the probiotic potential of bacteroides spp. within one health paradigm. Probiotics Antimicrob Proteins. (2025) 17:681–704. doi: 10.1007/s12602-024-10370-9, PMID: 39377977 PMC11925995

[31] Ku S Haque MA Jang MJ Ahn J Choe D Jeon JI. The role of bifidobacterium in longevity and the future of probiotics. Food Sci Biotechnol. (2024) 33:2097–110. doi: 10.1007/s10068-024-01631-y, PMID: 39130652 PMC11315853

[32] Kiliç GB Karahan AG. Identification of lactic acid bacteria isolated from the fecal samples of healthy humans and patients with dyspepsia, and determination of their ph, bile, and antibiotic tolerance properties. J Mol Microbiol Biotechnol. (2010) 18:220–9. doi: 10.1159/000319597 20668388

[33] Olm MR Dahan D Carter MM Merrill BD Yu FB Jain S. Robust variation in infant gut microbiome assembly across a spectrum of lifestyles. Science. (2022) 376:1220–3. doi: 10.1126/science.abj2972, PMID: 35679413 PMC9894631

[34] Mayer EA Nance K Chen S. The gut-brain axis. Annu Rev Med. (2022) 73:439–53. doi: 10.1146/annurev-med-042320-014032, PMID: 34669431

[35] Laue HE Coker MO Madan JC. The developing microbiome from birth to 3 years: the gut-brain axis and neurodevelopmental outcomes. Front Pediatr. (2022) 10:815885. doi: 10.3389/fped.2022.815885, PMID: 35321011 PMC8936143

[36] Wang M Zhang Z Liu Y Jian E Ye P Jiang H. Research trends between childhood obesity and gut microbiota: a bibliometric analysis (2002-2023). Front Microbiol. (2024) 15:1461306. doi: 10.3389/fmicb.2024.1461306, PMID: 39397792 PMC11466780

[37] Sanchez M Darimont C Drapeau V Emady-Azar S Lepage M Rezzonico E. Effect of *Lactobacillus rhamnosus* CGMCC1.3724 supplementation on weight loss and maintenance in obese men and women. Br J Nutr. (2014) 111:1507–19. doi: 10.1017/S0007114513003875, PMID: 24299712

[38] Li H Capital Institute of Pediatrics, Coordinating Study Group of Nine Cities on the Physical Growth and Development of Children. Growth standardized values and curves based on weight, length/height and head circumference for Chinese children under 7 years of age. Zhonghua Er Ke Za Zhi Chin J Pediatr. (2009) 47:173–8. doi: 10.3760/cma.j.issn.0578-1310.2009.03.005 19573429

[39] Cole TJ Green PJ. Smoothing reference centile curves: the LMS method and penalized likelihood. Stat Med. (1992) 11:1305–19. doi: 10.1002/sim.4780111005, PMID: 1518992

[40] Rito AI Buoncristiano M Spinelli A Salanave B Kunešová M Hejgaard T. Association between characteristics at birth, breastfeeding and obesity in 22 countries: the WHO European childhood obesity surveillance initiative – COSI 2015/2017. Obes Facts. (2019) 12:226–43. doi: 10.1159/000500425, PMID: 31030194 PMC6547266

[41] Starling AP Brinton JT Glueck DH Shapiro AL Harrod CS Lynch AM. Associations of maternal BMI and gestational weight gain with neonatal adiposity in the healthy start study. Am J Clin Nutr. (2015) 101:302–9. doi: 10.3945/ajcn.114.094946, PMID: 25646327 PMC4307203

[42] He JR Ramakrishnan R Wei XL Lu JH Lu MS Xiao WQ. Fetal growth at different gestational periods and risk of impaired childhood growth, low childhood weight and obesity: a prospective birth cohort study. BJOG Int J Obstet Gynaecol. (2021) 128:1615–24. doi: 10.1111/1471-0528.16698, PMID: 33690938

[43] Larqué E Labayen I Flodmark CE Lissau I Czernin S Moreno LA. From conception to infancy – early risk factors for childhood obesity. Nat Rev Endocrinol. (2019) 15:456–78. doi: 10.1038/s41574-019-0219-1, PMID: 31270440

[44] Pringle KG Lee YQ Weatherall L Keogh L Diehm C Roberts CT. Influence of maternal adiposity, preterm birth and birth weight centiles on early childhood obesity in an indigenous Australian pregnancy-through-to-early-childhood cohort study. J Dev Orig Health Dis. (2019) 10:39–47. doi: 10.1017/S2040174418000302, PMID: 29764530

[45] Farooq A Basterfield L Adamson AJ Pearce MS Hughes AR Janssen X. Moderate-to-vigorous intensity physical activity and sedentary behaviour across childhood and adolescence, and their combined relationship with obesity risk: a multi-trajectory analysis. Int J Environ Res Public Health. (2021) 18:7421. doi: 10.3390/ijerph18147421, PMID: 34299872 PMC8305282

[46] Vasylyeva TL Barche A Chennasamudram SP Sheehan C Singh R Okogbo ME. Obesity in prematurely born children and adolescents: follow up in pediatric clinic. Nutr J. (2013) 12:150. doi: 10.1186/1475-2891-12-150, PMID: 24252330 PMC3842808

[47] Williams AS Ge B Petroski G Kruse RL McElroy JA Koopman RJ. Socioeconomic status and other factors associated with childhood obesity. J Am Board Fam Med JABFM. (2018) 31:514–21. doi: 10.3122/jabfm.2018.04.170261, PMID: 29986976 PMC9118515

[48] Dennison BA Erb TA Jenkins PL. Television viewing and Television in Bedroom Associated with Overweight Risk among low-Income Preschool Children. Pediatrics. (2002) 109:1028–35. doi: 10.1542/peds.109.6.1028, PMID: 12042539

[49] Chen AC Fang TJ Ho HH Chen JF Kuo YW Huang YY. A multi-strain probiotic blend reshaped obesity-related gut dysbiosis and improved lipid metabolism in obese children. Front Nutr. (2022) 9:922993. doi: 10.3389/fnut.2022.922993, PMID: 35990345 PMC9386160

[50] Khongtan S Sivamaruthi BS Thangaleela S Kesika P Bharathi M Sirilun S. The influence of probiotic supplementation on the obesity indexes, neuroinflammatory and oxidative stress markers, gut microbial diversity, and working memory in obese thai children. Food Secur. (2023) 12:3890. doi: 10.3390/foods12213890, PMID: 37959009 PMC10648263

[51] Heymsfield SB Wadden TA. Mechanisms, pathophysiology, and Management of Obesity. N Engl J Med. (2017) 376:1492. doi: 10.1056/NEJMc1701944 28402780

[52] Huang Y Lu Z Liu F Lane JA Chen J Huang Q. Osteopontin associated *Bifidobacterium bifidum* microencapsulation modulates infant fecal fermentation and gut microbiota development. Food Res Int Ott Ont. (2024) 197:115211. doi: 10.1016/j.foodres.2024.115211, PMID: 39593296

[53] Walsh C Lane JA van Sinderen D Hickey RM. Human milk oligosaccharide-sharing by a consortium of infant derived Bifidobacterium species. Sci Rep. (2022) 12:4143. doi: 10.1038/s41598-022-07904-y, PMID: 35264656 PMC8907170

[54] Ohland CL Kish L Bell H Thiesen A Hotte N Pankiv E. Effects of *Lactobacillus helveticus* on murine behavior are dependent on diet and genotype and correlate with alterations in the gut microbiome. Psychoneuroendocrinology. (2013) 38:1738–47. doi: 10.1016/j.psyneuen.2013.02.008, PMID: 23566632

[55] Bernini LJ Simão ANC Alfieri DF Lozovoy MAB Mari NL de Souza CHB. Beneficial effects of *Bifidobacterium lactis* on lipid profile and cytokines in patients with metabolic syndrome: a randomized trial. Effects of probiotics on metabolic syndrome. Nutr Burbank Los Angel Cty Calif. (2016) 32:716–9. doi: 10.1016/j.nut.2015.11.001 27126957

[56] Sanchez M Darimont C Panahi S Drapeau V Marette A Taylor VH. Effects of a diet-based weight-reducing program with probiotic supplementation on satiety efficiency, eating behaviour traits, and psychosocial Behaviours in obese individuals. Nutrients. (2017) 9:284. doi: 10.3390/nu9030284, PMID: 28294985 PMC5372947

[57] Gallou-Kabani C Gabory A Tost J Karimi M Mayeur S Lesage J. Sex- and diet-specific changes of imprinted gene expression and DNA methylation in mouse placenta under a high-fat diet. PLoS One. (2010) 5:e14398. doi: 10.1371/journal.pone.0014398, PMID: 21200436 PMC3006175

[58] Stapleton S Welch G DiBerardo L Freeman LR. Sex differences in a mouse model of diet-induced obesity: the role of the gut microbiome. Res Sq. (2023). 5:rs.3.rs-3496738. doi: 10.1186/s13293-023-00580-1 PMC10782710 38200579

[59] He P Yu L Tian F Chen W Zhang H Zhai Q. Effects of probiotics on preterm infant gut microbiota across populations: a systematic review and meta-analysis. Adv Nutr Bethesda Md. (2024) 15:100233. doi: 10.1016/j.advnut.2024.100233, PMID: 38908894 PMC11251410

[60] Lai MY Chang YH Lee CC. Neonatal microbiome outcomes study group (NEMO). The impact of gut microbiota on morbidities in preterm infants. Kaohsiung J Med Sci. (2024) 40:780–8. doi: 10.1002/kjm2.12878, PMID: 39073226 PMC11895645

[61] Han J Jiang Y Huang J Zhang Y Zhang Y Zhang Y. Postnatal growth of preterm infants during the first two years of life: catch-up growth accompanied by risk of overweight. Ital J Pediatr. (2021) 47:66. doi: 10.1186/s13052-021-01019-2, PMID: 33726805 PMC7968173

[62] Hirschler V Edit S Miorin C Guntsche Z Maldonado N Garcia C. Association between high birth weight and later central obesity in 9-year-old schoolchildren. Metab Syndr Relat Disord. (2021) 19:213–7. doi: 10.1089/met.2020.0127, PMID: 33290153

[63] Mazziotta C Tognon M Martini F Torreggiani E Rotondo JC. Probiotics mechanism of action on immune cells and beneficial effects on human health. Cells. (2023) 12:184. doi: 10.3390/cells12010184 36611977 PMC9818925

[64] Bock PM Martins AF Schaan BD. Understanding how pre- and probiotics affect the gut microbiome and metabolic health. Am J Physiol Endocrinol Metab. (2024) 327:E89–E102. doi: 10.1152/ajpendo.00054.2024, PMID: 38809510

[65] Wang G Xie L Huang Z Xie J. Recent advances in polysaccharide biomodification by microbial fermentation: production, properties, bioactivities, and mechanisms. Crit Rev Food Sci Nutr. (2024) 64:12999–3023. doi: 10.1080/10408398.2023.2259461, PMID: 37740706

[66] Cristofori F Dargenio VN Dargenio C Miniello VL Barone M Francavilla R. Anti-inflammatory and immunomodulatory effects of probiotics in gut inflammation: a door to the body. Front Immunol. (2021) 12:578386. doi: 10.3389/fimmu.2021.578386, PMID: 33717063 PMC7953067

[67] Bermúdez-Humarán LG Chassaing B Langella P. Exploring the interaction and impact of probiotic and commensal bacteria on vitamins, minerals and short chain fatty acids metabolism. Microb Cell Factories. (2024) 23:172. doi: 10.1186/s12934-024-02449-3, PMID: 38867272 PMC11167913

[68] Li HY Zhou DD Gan RY Huang SY Zhao CN Shang A. Effects and mechanisms of probiotics, prebiotics, synbiotics, and postbiotics on metabolic diseases targeting gut microbiota: a narrative review. Nutrients. (2021) 13:3211. doi: 10.3390/nu13093211, PMID: 34579087 PMC8470858

[69] Alard J Lehrter V Rhimi M Mangin I Peucelle V Abraham AL. Beneficial metabolic effects of selected probiotics on diet-induced obesity and insulin resistance in mice are associated with improvement of dysbiotic gut microbiota. Environ Microbiol. (2016) 18:1484–97. doi: 10.1111/1462-2920.13181, PMID: 26689997

[70] Vähämiko S Laiho A Lund R Isolauri E Salminen S Laitinen K. The impact of probiotic supplementation during pregnancy on DNA methylation of obesity-related genes in mothers and their children. Eur J Nutr. (2019) 58:367–77. doi: 10.1007/s00394-017-1601-1, PMID: 29299736

