# Supplementary material for: Early supplement of probiotics reduces the risk of obesity among preschool children: a real-world observational study
Source: Front Nutr. 2025 May 15;12:1597894. doi: 10.3389/fnut.2025.1597894 (PMC12121508; doi:10.3389/fnut.2025.1597894)
Supplement: Supplementary file 1 [file Table_1.docx]

Supplementary Material

**Table S1.** The probiotic strains contained in each product

| **Product** | **Probiotic strains** | **Estimated Daily Dose** | **CFU/Day (approx.)** | **Carrier Matrix** | **Proportion of use ^a^** |
| --- | --- | --- | --- | --- | --- |
| Product A | *Bifidobacterium longum* subsp*. infantis* R0033,  *Bifidobacterium bifidum* R0071,  *Lactobacillus helveticus* R0052 | 3g | *Bifidobacterium spp.* : 5.7×10^8^,  *L. Helveticus* :  1.9×10^10^ | Powder in sachets | 35.6% |
| Product B | *Lacticaseibacillus rhamnosus* HN001*,*  *Bifidobacterium animalis subsp. lactis* HN019 | 3g | Total : 4.1×10^9^ | Powder in sachets | 19.2% |

a: Estimation was conducted among the study subjects taking probiotics included in the analysis.

**Table S2.** Association between variables and weight status using Chi-square tests

| **Variables** | **Expected frequency** | | | **Test type** | **χ2** | ***p*-value** |
| --- | --- | --- | --- | --- | --- | --- |
|  | **Normal weight** | **Overweight** | **Obesity** |  |  |  |
| ***Gender*** | | | | Chi-Square | 335.51 | ＜0.001 |
| Male | 13474 | 2295 | 735 |  |  |  |
| Female | 11990 | 2042 | 654 |  |  |  |
| ***Age (years)*** | | | | Chi-Square | 18.21 | 0.006 |
| 3~4 | 6156 | 1048 | 336 |  |  |  |
| 4~5 | 8243 | 1404 | 450 |  |  |  |
| 5~6 | 8118 | 1383 | 443 |  |  |  |
| 6~7 | 2947 | 502 | 161 |  |  |  |
| ***Gestational Age (weeks)*** | | | | Chi-Square | 2.69 | 0.261 |
| <37 | 1834 | 312 | 100 |  |  |  |
| ≥37 | 23630 | 4025 | 1289 |  |  |  |
| ***Child birth weight(g)*** | | | | Chi-Square | 82.71 | ＜0.001 |
| ＜2500 | 1738 | 296 | 95 |  |  |  |
| 2500~4000 | 23067 | 3929 | 1258 |  |  |  |
| >4000 | 659 | 112 | 36 |  |  |  |
| ***Maternal age at conception (years)*** | | | | Chi-Square | 11.91 | 0.003 |
| <35 | 22864 | 3894 | 1247 |  |  |  |
| ≥35 | 2600 | 443 | 142 |  |  |  |
| ***Maternal pre-pregnancy BMI (kg/m***2***)*** | | | | Chi-Square | 210.25 | ＜0.001 |
| <18.5 | 4987 | 849 | 272 |  |  |  |
| 18.5~23.9 | 17965 | 3060 | 980 |  |  |  |
| ≥24 | 2511 | 428 | 137 |  |  |  |

**Table S2.** Association between variables and weight status using Chi-square tests (continued)

| **Variables** | **Expected frequency** | | | **Test type** | **χ2** | ***p*-value** |
| --- | --- | --- | --- | --- | --- | --- |
|  | **Normal weight** | **Overweight** | **Obesity** |  |  |  |
| ***Maternal education level*** | | | | Chi-Square | 17.03 | 0.008 |
| ≤Middle school | 2855 | 486 | 156 |  |  |  |
| High school | 4367 | 744 | 238 |  |  |  |
| College | 17221 | 2933 | 939 |  |  |  |
| ≥Postgraduate | 1021 | 174 | 56 |  |  |  |
| ***Paternal education level*** | | | | Chi-Square | 36.86 | ＜0.001 |
| ≤Middle school | 2559 | 436 | 140 |  |  |  |
| High school | 4532 | 772 | 247 |  |  |  |
| College | 16846 | 2869 | 919 |  |  |  |
| ≥Postgraduate | 1528 | 260 | 83 |  |  |  |
| ***Household income (RMB/month)*** | | | | Chi-Square | 2.68 | 0.848 |
| ≤10,000 | 3821 | 651 | 208 |  |  |  |
| 10,001~20,000 | 7923 | 1349 | 432 |  |  |  |
| 20,001~30,000 | 5401 | 920 | 295 |  |  |  |
| >30,000 | 8319 | 1417 | 454 |  |  |  |
| ***Feeding pattern*** | | | | Chi-Square | 1.01 | 0.985 |
| Breastfeeding | 14232 | 2424 | 776 |  |  |  |
| Bottle feeding | 2652 | 452 | 145 |  |  |  |
| Mixed feeding | 8486 | 1445 | 463 |  |  |  |
| Not clear | 95 | 16 | 5 |  |  |  |
| ***Child physical activity frequency (days/week)*** | | | | Chi-Square | 2.19 | 0.902 |
| 0 | 66 | 11 | 4 |  |  |  |
| 1~2 | 5522 | 941 | 301 |  |  |  |
| 3~6 | 8602 | 1465 | 469 |  |  |  |
| 7 | 11274 | 1920 | 615 |  |  |  |

**Table S2.** Association between variables and weight status using Chi-square tests (continued)

| **Variables** | **Expected frequency** | | | **Test type** | **χ2** | ***p*-value** |
| --- | --- | --- | --- | --- | --- | --- |
|  | **Normal weight** | **Overweight** | **Obesity** |  |  |  |
| ***Electronic screen usage (minutes/day)*** | | | | Chi-Square | 17.74 | 0.007 |
| 0 | 6182 | 1053 | 337 |  |  |  |
| 1~30 | 11984 | 2041 | 654 |  |  |  |
| 31~60 | 5463 | 931 | 298 |  |  |  |
| >60 | 1834 | 312 | 100 |  |  |  |
| ***Whether taking probiotics*** | | | | Chi-Square | 20.21 | ＜0.001 |
| No | 5515 | 939 | 301 |  |  |  |
| Yes | 19949 | 3398 | 1088 |  |  |  |
| ***Probiotic initiation time (months)*** | | | | Chi-Square | 29.67 | ＜0.001 |
| No | 5515 | 939 | 301 |  |  |  |
| 0-6 | 4112 | 700 | 224 |  |  |  |
| 6-12 | 6210 | 1058 | 339 |  |  |  |
| 12-36 | 6268 | 1068 | 342 |  |  |  |
| Not clear | 3358 | 572 | 183 |  |  |  |
| ***Probiotic cumulative duration (months)*** | | | | Chi-Square | 32.02 | ＜0.001 |
| No | 5515 | 939 | 301 |  |  |  |
| <1 | 6936 | 1181 | 378 |  |  |  |
| 1-3 | 3835 | 653 | 209 |  |  |  |
| 3-6 | 2208 | 376 | 120 |  |  |  |
| >6 | 3283 | 559 | 179 |  |  |  |
| Not clear | 3688 | 628 | 201 |  |  |  |
| ***Probiotic product*** | | | | Chi-Square | 23.42 | 0.003 |
| No | 5515 | 939 | 301 |  |  |  |
| Product A | 7104 | 1210 | 387 |  |  |  |
| Product B | 3833 | 653 | 209 |  |  |  |
| Other strains | 2886 | 492 | 157 |  |  |  |
| Not clear | 6126 | 1043 | 334 |  |  |  |

**Table S3.** Simplified logistic regression: outcome binary variable weight status

(overweight vs. normal weight)

| **Probiotic supplement status** | **AOR (95% CI) ^a^** | ***p*-Value** |
| --- | --- | --- |
| ***Whether taking*** |  | |
| No | ref |  |
| Yes | 0.88 (0.82, 0.96) | 0.002 |
| ***Initiation time (months)*** |  | |
| No | ref |  |
| 0-6 | 0.95 (0.85, 1.05) | 0.312 |
| 6-12 | 0.88 (0.80, 0.97) | 0.008 |
| 12-36 | 0.82 (0.75, 0.91) | <0.001 |
| Not clear | 0.94 (0.84, 1.05) | 0.247 |
| ***Cumulative duration (months)*** |  | |
| No | ref |  |
| <1 | 0.82 (0.75, 0.91) | <0.001 |
| 1-3 | 0.91 (0.82, 1.01) | 0.080 |
| 3-6 | 0.91 (0.80, 1.03) | 0.140 |
| >6 | 0.91 (0.81, 1.02) | 0.092 |
| Not clear | 0.94 (0.84, 1.04) | 0.222 |
| ***Probiotic product*** |  | |
| No | ref |  |
| Product A | 0.88 (0.80, 0.96) | 0.005 |
| Product B | 0.91 (0.81, 1.01) | 0.075 |
| Other products | 0.85 (0.76, 0.96) | 0.009 |
| Not clear | 0.89 (0.81, 0.98) | 0.019 |

a: Adjusted for children’s gender and age, gestational age birth weight , maternal age at conception and pre-pregnancy BMI, parental education level and marital status, household income, the child’s feeding pattern, physical activity frequency and electronic screen usage in models. ref: reference.

**Table S4.** Simplified logistic regression: outcome binary variable weight status

(obesity vs. normal weight)

| **Probiotic supplement status** | **AOR (95% CI) ^a^** | ***p*-Value** |
| --- | --- | --- |
| ***Whether taking*** |  | |
| No | ref |  |
| Yes | 0.82 (0.72, 0.93) | 0.002 |
| ***Initiation time (months)*** |  | |
| No | ref |  |
| 0-6 | 0.76 (0.64, 0.92) | 0.004 |
| 6-12 | 0.87 (0.75, 1.02) | 0.089 |
| 12-36 | 0.80 (0.69, 0.94) | 0.007 |
| Not clear | 0.82 (0.68, 0.99) | 0.044 |
| ***Cumulative duration (months)*** |  | |
| No | ref |  |
| <1 | 0.80 (0.69, 0.94) | 0.005 |
| 1-3 | 0.79 (0.66, 0.95) | 0.011 |
| 3-6 | 0.79 (0.63, 0.99) | 0.039 |
| >6 | 0.83 (0.69, 1.01) | 0.058 |
| Not clear | 0.90 (0.75, 1.07) | 0.225 |
| ***Probiotic product*** |  | |
| No | ref |  |
| Product A | 0.85 (0.73, 0.99) | 0.031 |
| Product B | 0.84 (0.70, 1.01) | 0.065 |
| Other products | 0.76 (0.62, 0.93) | 0.007 |
| Not clear | 0.81 (0.69, 0.95) | 0.009 |

a: Adjusted for children’s gender and age, gestational age birth weight , maternal age at conception and pre-pregnancy BMI, parental education level and marital status, household income, the child’s feeding pattern, physical activity frequency and electronic screen usage in models. ref: reference.

**Table S5.** Bayesian multinomial logistic regression: outcome trichotomous variable weight status

(overweight or obesity vs. normal weight)

| **Probiotic supplementation** | **Overweight (vs. Normal weight)** | | **Obesity (vs. Normal weight)** | |
| --- | --- | --- | --- | --- |
|  | **AOR (95% CI) ^a^** | **pd (%)** | **AOR (95% CI) ^a^** | **pd (%)** |
| ***Whether taking*** | | | | |
| No | ref |  | ref |  |
| Yes | 0.88 (0.82, 0.95) | 99.95 | 0.82 (0.72, 0.92) | 99.95 |
| ***Initiation time (months)*** | | | | |
| No | ref |  | ref |  |
| 0-6 | 0.95 (0.85, 1.05) | 85.35 | 0.76 (0.64, 0.91) | 99.88 |
| 6-12 | 0.88 (0.80, 0.97) | 99.85 | 0.87 (0.74, 1.01) | 96.70 |
| 12-36 | 0.82 (0.75, 0.91) | 99.98 | 0.80 (0.68, 0.94) | 99.67 |
| Not clear | 0.93 (0.84, 1.04) | 87.70 | 0.82 (0.68, 1.00) | 97.75 |
| ***Cumulative duration (months)*** | | | | |
| No | ref |  | ref |  |
| <1 | 0.82 (0.75, 0.91) | 100 | 0.80 (0.68, 0.93) | 99.70 |
| 1-3 | 0.91 (0.82, 1.01) | 96.50 | 0.78 (0.65, 0.94) | 99.58 |
| 3-6 | 0.91 (0.80, 1.02) | 93.53 | 0.78 (0.63, 0.96) | 99.08 |
| >6 | 0.91 (0.81, 1.02) | 95.33 | 0.84 (0.69, 1.00) | 97.15 |
| Not clear | 0.94 (0.84, 1.04) | 88.45 | 0.88 (0.74, 1.06) | 91.12 |
| ***Probiotic product*** | | | | |
| No | ref |  | ref |  |
| Product A | 0.88 (0.80, 0.96) | 99.62 | 0.84 (0.72, 0.99) | 98.30 |
| Product B | 0.91 (0.82, 1.01) | 95.62 | 0.84 (0.70, 1.01) | 96.73 |
| Other products | 0.85 (0.75, 0.96) | 99.60 | 0.75 (0.61, 0.92) | 99.85 |
| Not clear | 0.89 (0.81, 0.98) | 99.20 | 0.80 (0.68, 0.95) | 99.48 |

a: Adjusted for children’s gender and age, gestational age birth weight, maternal age at conception and pre-pregnancy BMI, parental education level and marital status, household income, the child’s feeding pattern, physical activity frequency and electronic screen usage. A random effect for kindergarten was included in the models. pd: probability of direction. ref: reference.

**Project on Establishment and Tracking of Health Records for Preschool Children in Longhua District, Shenzhen**

**Informed Consent**

Dear Parents:

Shalom!

The "Pre-school Children Health Record Establishment and Tracking Project" is a public welfare project jointly undertaken by the Longhua District Health Bureau of Shenzhen, the Longhua District Maternal and Child Health Hospital, and the School of Public Health at Sun Yat-sen University. The project aims to establish psychological and behavioral health records for every child in the Longhua District's kindergartens and follow up with them. It will promptly provide you with relevant information and offer targeted guidance, suggestions, or treatment plans. This project is of great significance in promoting the physical and mental health of your child, and it also provides important reference value for the government in formulating policies and implementing public measures related to preschool children's health.

We will gather information about your child and family through a questionnaire. Additionally, we will obtain health check-up information for your child at school (including height, weight, and blood pressure). To ensure the accuracy and reliability of your child's health records, please fill out the form carefully and truthfully. There are no standard answers to any of the questions; there is no right or wrong, good or bad. Please choose the most suitable answer based on your actual situation and genuine feelings. This information will only be used by the government and universities to provide interventions and guidance for promoting children's health. We solemnly promise that all the information you provide will be strictly confidential and will not be disclosed to any organization or individual.

Thank you very much for taking the time to fill in this form! I sincerely wish your family health and children grow up healthily!

I have read the above information and voluntarily fill in the questionnaire of "Pre-school Children Health Record Establishment and Tracking Project".

Parent signature:______________

Date of filling:________________
